# MRI-based Radiomics nomogram to detect primary rectal cancer with synchronous liver metastases

**DOI:** 10.1038/s41598-019-39651-y

**Published:** 2019-03-04

**Authors:** Zhenyu Shu, Songhua Fang, Zhongxiang Ding, Dewang Mao, Rui Cai, Yuanjun Chen, Peipei Pang, Xiangyang Gong

**Affiliations:** 1 Department of Radiology, Zhejiang Provincial People’s Hospital, Affiliated People’s Hospital of Hangzhou Medical College, Hangzhou, China; 2 Department of Anorectal, Zhejiang Provincial People’s Hospital, Affiliated People’s Hospital of Hangzhou Medical College, Hangzhou, China; 3 GE Healthcare China, Shanghai, China

## Abstract

Synchronous liver metastasis (SLM) remains a major challenge for rectal cancer. Early detection of SLM is a key factor to improve the survival rate of rectal cancer. In this radiomics study, we predicted the SLM based on the radiomics of primary rectal cancer. A total of 328 radiomics features were extracted from the T2WI images of 194 patients. The least absolute shrinkage and selection operator (LASSO) regression was used to reduce the feature dimension and to construct the radiomics signature. after LASSO, principal component analysis (PCA) was used to sort the features of the surplus characteristics, and selected the features of the total contribution of 85%. Then the prediction model was built by linear regression, and the decision curve analysis was used to judge the net benefit of LASSO and PCA. In addition, we used two independent cohorts for training (n = 135) and validation (n = 159). We found that the model based on LASSO dimensionality construction had the maximum net benefit (in the training set (AUC [95% confidence interval], 0.857 [0.787–0.912]) and in the validation set (0.834 [0.714–0.918]). The radiomics nomogram combined with clinical risk factors and LASSO features showed a good predictive performance in the training set (0.921 [0.862–0.961]) and validation set (0.912 [0.809–0.97]). Our study indicated that radiomics based on primary rectal cancer could provide a non-invasive way to predict the risk of SLM in clinical practice.

## Introduction

Rectal cancer is one of the most common malignant tumors of digestive tract with an increasing morbidity in the past few years^1^, and 15~20% of the patients with rectal cancer have liver metastases at the time of diagnosis, which is defined as synchronous liver metastasis (SLM)^2^. SLM is a leading cause of mortality in patients with rectal cancer^3^ and also an important prognostic factor. Surgical resection of SLM and neoadjuvant therapy have been shown to improve the progression-free survivals of patients with rectal cancer and SLM^4,5^. Therefore, identification of the risk of SLM in patients with rectal cancer is crucial for personalized treatment planning.

Computed Tomography (CT) is the modality of choice based on abdominal for the preoperative evaluation of SLM^6^, but the ability of detection and differentiation of SLM are limited by the lesion sizes and overlapping CT imaging patterns. CT is insensitive to detect lesions less than 10 mm and to distinguish SLMs from other small hepatic lesions, such as hemangiomas and cysts^7^. The concern of radiation doses also limits the wide use of CT as a routine method for detecting SLM. T2-weighted imaging (T2WI) in magnetic resonance imaging (MRI) is an important clinical imaging technique, which can provide high signal-to-noise ratio, spatial resolution and soft tissue contrast images for the tumor structures, and has been widely used for tumor grading based on morphological changes^8,9^. In a morphological study of primary rectal cancer by Taylor *et al*., tumor responses can contribute to more than 70% of tumor volumetric changes detected in T_2_WI^10^. However, conventional morphological assessment is insensitive to predict the risk of SLM.

Radiomics is a recently emerging field in radiology by quantify imaging data with the aid of advanced image processing techniques^11,12^, including a high-throughput analysis and features selection to build a signature for a complete characterization of the tumors^13–15^. A study showed that the texture analysis of liver parenchyma extracted from CT images could predict survivals of colorectal cancer patient, in other words, it suggested that the different prognosis of colorectal cancer patients could be reflected in the texture features of liver CT image^16^. In this study, we hypothesized that the imaging biomarkers established by radiomics features extracted from the primary rectal cancer lesion can non-invasively identify high-risk rectal cancers, which have higher possibility of SLM. To our knowledge, there has been no radiomics-based study to rectal cancer with high risk of SLM.

Hence, in this study, we sought to develop and validate a radiomics nomogram that would incorporate a radiomics signature based on primary rectal cancer for the preoperative prediction of SLM.

## Materials and Methods

### General Information

This retrospective study was approved by the ethics committee of Zhejiang Provincial People’s hospital and informed consents was obtained from all patients. The research method was carried out in accordance with the relevant guidelines and regulations. The surgical and radiological database was retrospectively reviewed between 2012 and 2017. The inclusion criteria were as follows: (a) patients with histopathologically proved rectal cancer, (b) no history of previous or coexisting other malignancy, (c) patients who underwent preoperative high-resolution rectal MRI for local staging of rectal cancer. (d) patients who underwent preoperative abdominopelvic CT or liver MRI for liver metastases. Among these patients, 88 patients were excluded for the following reasons: (1) patients underwent therapy (radiotherapy, chemotherapy or chemoradiotherapy) before the baseline MRI examination (n = 24). (2) inflammatory diseases (n = 13), (3) poor imaging quality unqualified for image analysis (n = 15), (4) metachronous liver metastasis (n = 36). Eventually, a total of 194 consecutive patients were included in our study. Among them, 75 patients were diagnosed with liver metastases based on the clinicopathological results. All patients were randomly divided into the training (n = 135) and validation (n = 59) sets according to 7:3.

### MRI image acquisition

All enrolled patients were scanned by a 3.0T MR (Discovery MR 750, GE Healthcare, Waukesha, WI, USA). Axial T2WI Fast Recovery Fast Spin Echo (FR-FSE) sequence was used, slice thickness: 3 mm; interval: 0.3 mm; TR/TE: 3500∼5000 ms/115 ms; FOV: 18 cm × 18 cm; pixel matrix: 256 × 256; ETL:20. Sagittal localizing T2-weighted fast spin-echo images were obtained, and the oblique axial and coronal T2-weighted fast spin-echo images were obtained orthogonal and parallel to the long axis of the rectal cancer. An axial T1-weighted fast gradient echo sequence was also performed. All sequences were performed without fat saturation.

### Region-of-interest segmentation and radiomic feature extraction

Tumor regions of interest (ROI) were semi-automatically segmented in the largest cross-sectional area in T2WI images using the ITK-SNAP software (www.itksnap.org). Radiomics features including Histogram, Formfactor, Gray-level co-occurrence matrix (GLCM), Run-length matrix (RLM) were calculated by AK software (Artificial Intelligence Kit V3.0.0.R, GE Healthcare). In total, 328 imaging features were extracted. in each patient before feature selection, including 32 histogram features, 8 formfactor features, 144 GLCM features and 144 RLM features, the details of features were described in Supplementary Data. Extracted texture features were standardized, which could remove the unit limits of the data of each feature. The dimension reduction was performed as follows: First, the analysis of variance (ANOVA) and Mann Whitney-U test (MW) were performed, and then the correlation test was calculated to reduce data redundancy. More information about the ROI segmentation procedure and radiomics feature extraction methodology can be found in the in the supplementary material.

### SLM-related feature selection and radiomics signature construction

The least absolute shrinkage and selection operator (LASSO) was used to further select the features, and then the principal component analysis (PCA) was used to sort the rest features by the importance and selected the overall contribution 85%. Finally, the multivariate logistic regression was used to build two models. Decision Curve Analysis (DCA) was performed to compare accuracy of single LASSO and combined PCA by calculating the net benefit with a spectrum of probability thresholds^17^. At last, the best dimension reduction method was used to build the radiomics signature and calculate the radiomics score (rad-score) for every patient. The predictive accuracy of the radiomics signature was quantified by the area under the receiver - operator characteristic (ROC) curve (AUC) in both the training and validation sets. More information about LASSO, PCA and DCA algorithm can be found in the Supplementary Data.

### Construction and Assessment of the radiomics nomogram for the rectal cancer with SLM

In the training data, univariate logistic regression was performed for each potential predictive variable, such as gender, age, primary tumor site, MRI tumor-stage (mT-stage), MRI lymph node-status (mLN-status) to select the independent clinical predictor. Multivariable logistic regression analysis combining the independent clinical risk factors and radiomic signature was applied to develop a diagnostic model for the SLM. A radiomics nomogram was then constructed on the basis of the multivariate logistic regression model. The calibration of the nomogram was assessed with a calibration curve. The Hosmer–Lemeshow test was performed to assess the goodness-of-fit of the nomogram. Furthermore, to evaluate the discriminatory ability of the nomogram, the ROC curves were then developed. A radiomics score was calculated for each patient in the validation set using the formula constructed in the training set. The calibration and the Hosmer–Lemeshow test were performed, and the AUC was calculated.

### The intra-observer and inter-observer agreement

The intra-observer and inter-observer agreements of feature extraction were evaluated by intra-class correlation coefficient (ICC). We initially chose 30 random T_2_WI images for ROI segmentation and feature extraction. The ROI segmentation was performed by two experienced radiologists independently. Intra-observer ICC was computed by comparing two extractions of reader A (with 10 years’ experience on abdominal MRI). Inter-observer ICC was computed by comparing the extraction of a second reader (reader B, with 15 years’ experience on abdominal MRI) and the first extraction of reader A. When the ICC was greater than 0.75, it was considered as good agreement, and the remaining image segmentation was performed by reader A.

### Statistical analysis

The LASSO logistic regression model was used with penalty parameter tuning that was conducted by 10-fold cross-validation based on minimum criteria. The backward stepwise selection was applied by means of the likelihood ratio test and took Akaike’s information criterion (AIC) as the stopping rule. The variables with univariate *P*-value < 0.05 were considered as candidate predictors in the multivariate logistic model.

The statistical analysis was conducted with R software (version 3.3.1). The reported statistical significance levels were all two-sided, with statistical significance set at 0.05. The multivariate binary logistic regression was done with the “rms” package. Nomogram construction and calibration plots were performed using the “rms” package. DCA was performed using the “dca.R.”. Other statistical analysis was performed with SPSS 17.0 and MedCalc15.2.2.

### Informed consent

Informed consent was obtained from all individual participants included in the study.

### Approval

A confirmed statement including all experimental protocols was approved by the institutional review board and informed consents.

### Accordance

A statement explicitly said that the methods were carried out in accordance with the relevant guidelines and regulations by the institutional review board.

## Results

### Patients’ characteristics

There was no statistic difference in variables (sex, age, primary tumor site, mT-stage and mLN-status) between the training and validation sets (Table 1, *P* > 0.05). There was statistic difference in variables (sex, mT-stage, mLN-status and rad-score) between SLM-positive and SLM-negative for training cohorts, which was analogous in the validation cohorts (Table 2, *P* < 0.05).

**Table 1:** Clinical characteristics of the Training and validation sets.

Variable	Training (n = 135)			validation (n = 59)		
		n	%	n	%	P value
Gender	male	96	71.1	46	78	0.321
	female	39	28.9	13	22	
Age(years)	**<60**	82	60.7	44	74.6	0.063
	**>60**	53	39.3	15	25.4	
Primary tumor site	Low-rectum	85	63	33	55.9	0.356
	Mid-rectum	50	37	26	44.1	
MRI T-stage	T1-2	70	51.9	31	52.5	0.517
	T3-4	65	48.1	28	47.5	
MRI LN-status	N0	43	31.9	16	27.1	0.51
	N1-2	92	68.1	43	72.9

**Table 2:** Clinical characteristics of the Training and validation sets for liver metastases.

Variable	Training (n = 135)				Validation (n = 59)		
	liver metastases (n = 52)		No liver metastases (n = 83)		liver metastases (n = 23)	No liver metastases (n = 36)	
		n (%)	n (%)	P value	n (%)	n (%)	P value
Gender	Male	40 (76.9)	56 (67.5)	0.238	20 (87)	26 (72.2)	0.183
	Female	12 (23.1)	27 (32.5)		3 (13)	10 (27.8)	
Age (years)	**>60**	31 (59.6)	51 (61.4)	0.832	16 (69.6)	28 (77.8)	0.480
	**<60**	21 (40.4)	32 (38.6)		7 (30.4)	8 (22.2)	
Primary tumor site	Low-rectum	35 (67.3)	50 (60.2)	0.408	13 (56.5)	20 (55.5)	0.942
	Mid-rectum	17 (32.7)	33 (39.8)		10 (43.5)	16 (44.5)	
MRI T-stage	T1-2	16 (30.8)	54 (65.1)	<0.0001	8 (34.8)	23 (63.9)	0.001
	T3-4	36 (69.2)	29 (34.9)		15 (65.2)	13 (36.1)	
MRI LN status	N0	13 (25)	30 (36.1)	0.176	5 (21.7)	11 (30.6)	0.458
	N1-2	39 (75)	53 (63.9)		18 (78.3)	25 (69.4)	
Rad score	−0.908 + 1.462		1.4777 + 1.7084	<0.0001	−1.1691 + 1.4255	1.0641 + 1.8279	<0.0001

### Inter-observer and intra-observer reproducibility of Radiomics feature extraction

The intra-observer ICC calculated based on two measurements of reader A ranged from 0.814 to 0.984. The inter-observer agreement between two readers ranged from 0.793 to 0.938. The results indicated favorable intra and inter-observer feature extraction reproducibility.

### Radiomics signature development and accuracy

**Figure 1:**
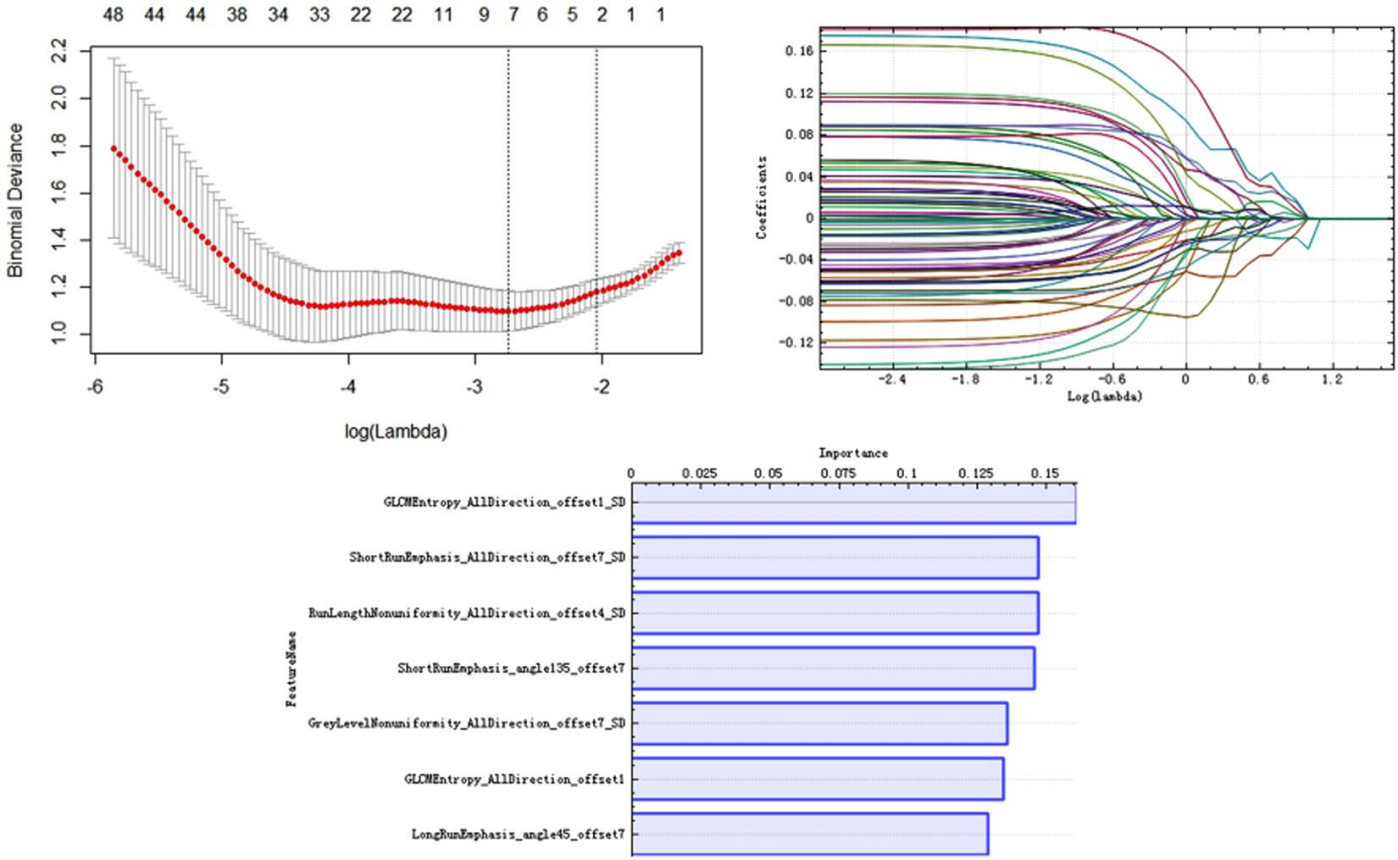
Texture feature Selection. (**A**) Tuning parameter (λ) selection in the LASSO model used ten-fold cross-validation via minimum criteria. The partial likelihood deviance was plotted versus log (λ). The dotted vertical lines were drawn at the optimal values using the minimum criteria and the 1-SE criteria. seven features were selected with the smallest binomial deviance. (**B**) LASSO coefficient profiles of texture features. Vertical line is drawn at the value selected using 10-fold cross-validation in log (λ) sequence and showed ten coefficients with non-zero were indicated. (**C**) The important sorting of PCA. Relative selection frequencies (%) of seven features by LASSO calculated from 1000 bootstrap samples.

Initially, 245 features were selected by ANOVA and MW test. After the spearman correlation analysis, there were 65 features remained (Fig. S2). The results of data dimension reduction with LASSO and PCA was showed in Fig. 1. LASSO with 7 most valuable variables and their coefficients were showed in Fig. 1A,B, and PCA with 6 features for an overall contribution 85% and the detail selection frequencies of 7 selected LASSO parameters (Fig. 1C). The multivariate logistic regression was used to build the models of single LASSO and combining PCA, The AUC of two models in validation set, LASSO was greater than PCA, but inversely in training set (Table S1).

**Figure 2:**
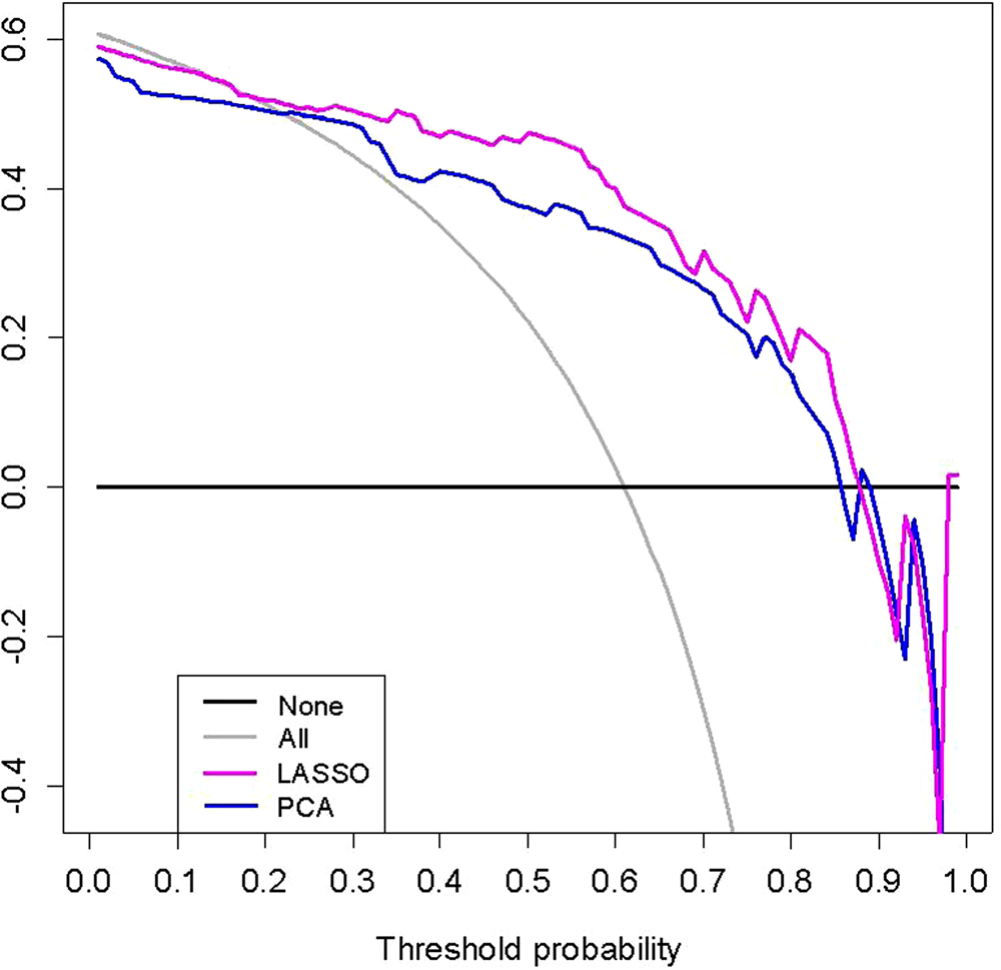
Decision curve analysis of method for data dimension reduction. The Y-axis represents the net benefit, which is calculated by summing the benefits (gaining true positives) and subtracting weighted harms (deleting false positives). The method is the best for feature selection if it has the highest net benefit.

**Figure 3:**
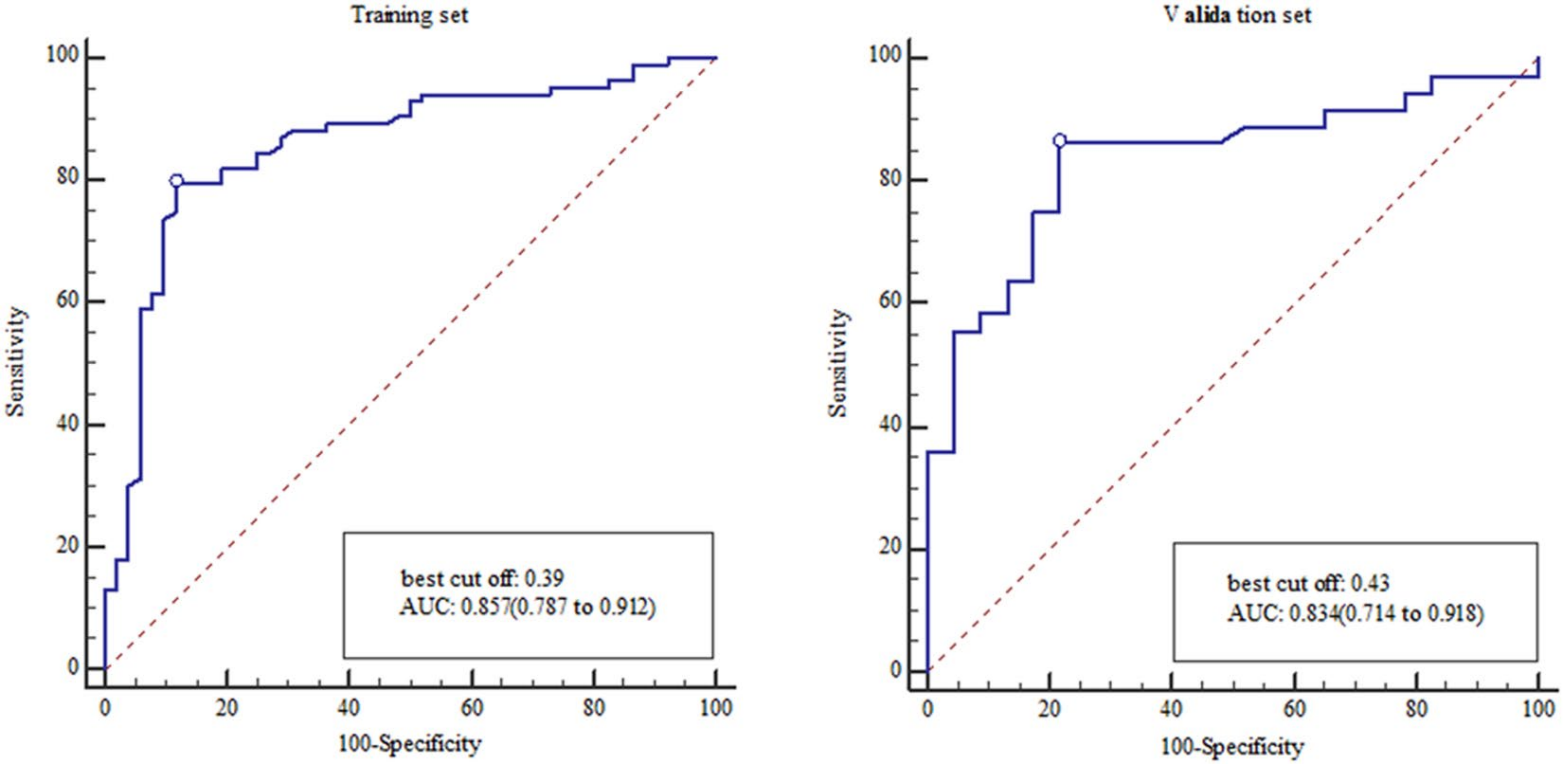
(**A**) The ROC curves effectiveness of the radiomics signature in the training set. (**B**) The ROC curves effectiveness of the radiomics signature in the validation set.

The DCA indicated that LASSO was the most valuable method in data dimension reduction, when the threshold probability for a radiomics feature was within a range from 0 to 0.92, and the LASSO added more net benefit than the “reduction all” or “reduction none” strategies (Fig. 2). So, in this paper, we used single LASSO models to build the rad-score by a linear combination of selected 7 features that were weighted by their respective LASSO coefficients (Rad-score calculation formula was provided in the supplementary material). The values of the selected 7 features in each patient was put into the formula, and then a risk score (defined as the rad-score) was obtained to reflect the risk of SLM. The radiomics signature showed favorable predictive efficacy, with an AUC of 0.857 [95% confidence interval (CI), 0.787 to 0.912] in the training set and 0.834 (95% CI, 0.714 to 0.918) in the validation set (Fig. 3A,B).

### Development and performance of Radiomics Nomogram

**Figure 4:**
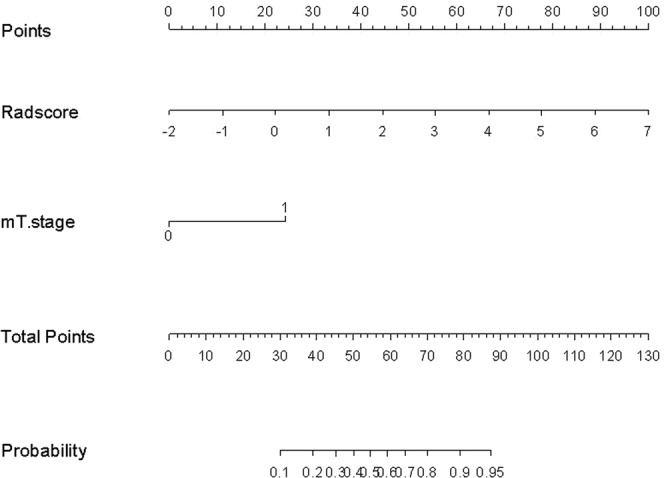
Radiomics nomogram to detect liver metastasis. The radiomics nomogram was developed in the training set, with the rad-score and mT.stage. In the nomogram, first, make a vertical line according to the value of rad-score to determine the corresponding value of points. In the same way, the points of mT.stage was also determined. then, total points were the sum of the two points above. finally, make a vertical line according to the value of total points to determine the probability of synchronous liver metastases.

**Figure 5:**
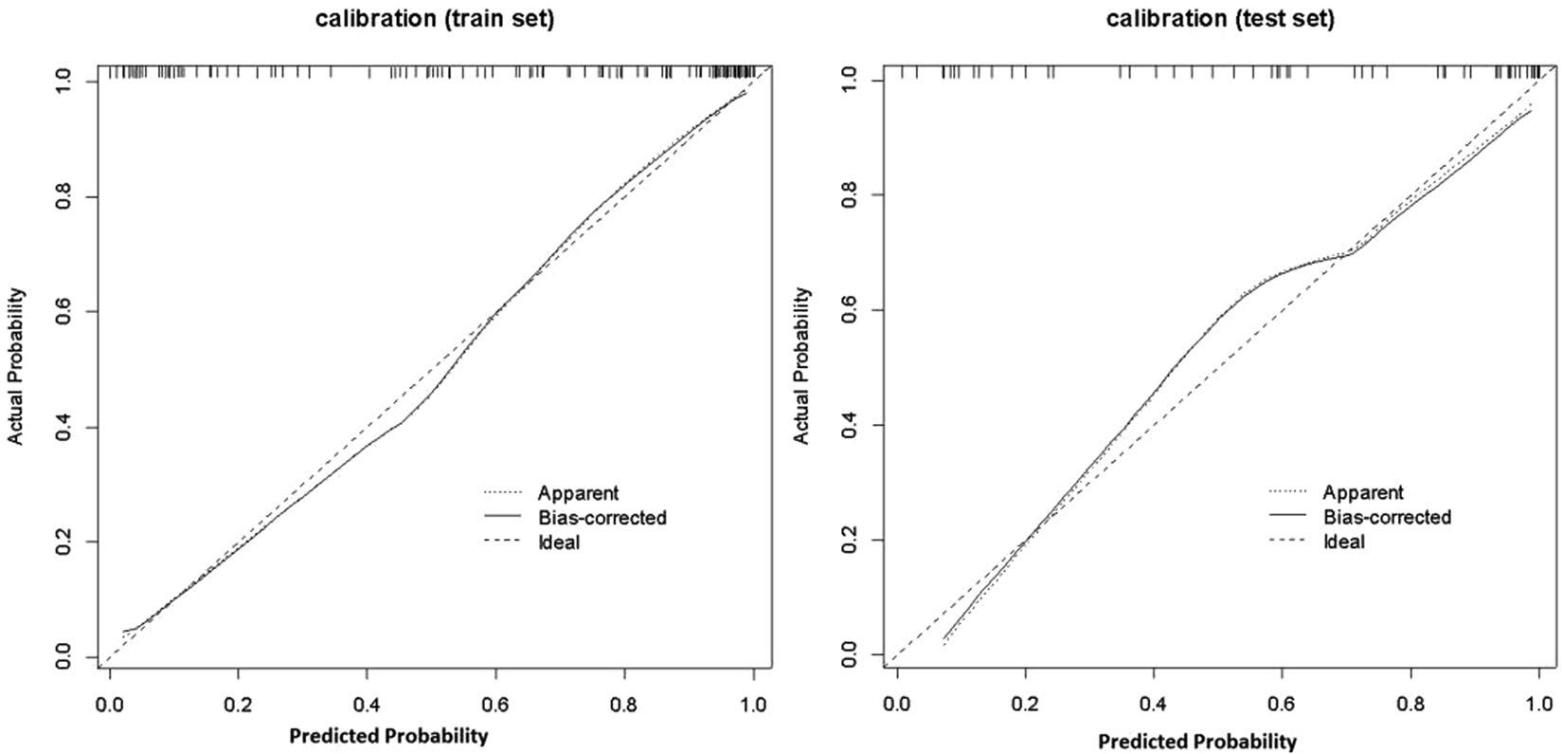
Calibration of the radiomics nomogram for synchronous liver metastasis in the training and validation sets (**A**,**B**). Dashed line was reference line where an ideal nomogram would lie. Dotted line was the performance of hybrid nomogram, while the solid line corrects for any bias in hybrid nomogram.

**Figure 6:**
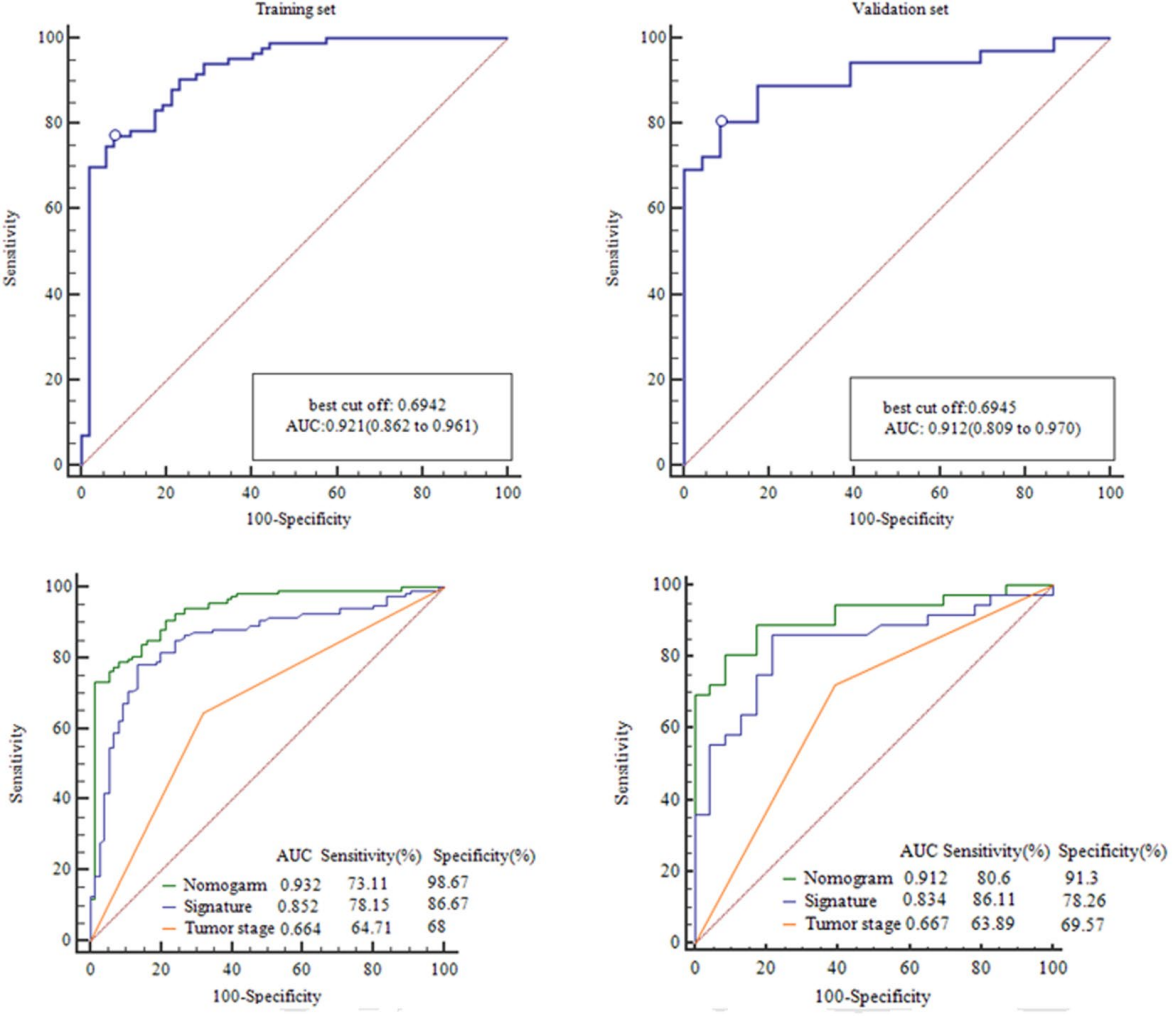
ROC curves of radiomics nomogram to detect the presence of liver Metastasis in the training set and validation set, respectively (**A**,**B**). ROC curves of MRI tumor stage, Radiomics nomogram, and Radiomics signature to detect the presence of rectal cancer with synchronous liver metastases in all patients and validation set, respectively (**C**,**D**).

**Figure 7:**
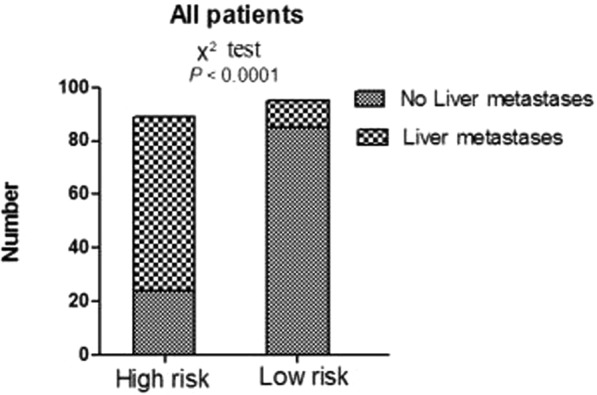
The risk classification performance of the nomogram in all patients. The possibility of SLM in high risk group was significantly higher than low risk group.

The radiomics score and mT-stage were identified as independent predictors of SLM in rectal cancer patients by a multivariate logistic regression model (Table 3). A radiomics nomogram incorporating these two predictors was constructed (Fig. 4**)**. A nonsignificant Hosmer - Lemeshow test statistic (*P* = 0.582) showed good calibration in the training set. The calibration curve of the nomogram showed good calibration in the training and validation sets (Fig. 5A,B). The detect accuracy of the nomogram for detecting SLM was 0.921 (95% CI, 0.862–0.961) in the training set and 0.912 (95% CI, 0.809–0.97) in the validation set, respectively (Fig. 6A,B). To compare the detection performance, the ROC curves based on all 194 patients were plotted for radiomics nomogram, radiomics signature and conventional mT-stage. The AUCs of the radiomics nomogram, radiomics signature and conventional mT-stage were 0.932 (95% CI, 0.887–0.963), 0.852 (95% CI, 0.794–0.899) and 0.664 (95% CI, 0.561–0.746), respectively (Fig. 6C). In addition, we also plotted the ROC curve in the validation set, The AUCs of the radiomics nomogram, radiomics signature and conventional mT-stage were 0.912 (95% CI, 0.809–0.970), 0.834 (95% CI, 0.714–0.918) and 0.667 (95% CI, 0.533–0.785), respectively (Fig. 6D). Therefore, the nomogram was superior to the radiomics signature and the mT-stage alone in detecting SLM. After getting the risk score of each patient from nomogram, we divided the patients into low-risk and high-risk groups according to the optimal cut off value (criterion 0.734) based on the maximum Youden index in the training set. The nomogram also showed good discriminatory ability in SLM, and the possibility of SLM in high risk group was significantly higher than low risk group (Fig. 7).

**Table 3:** Logistic regression analyses of predicting liver metastases: the final predictor for developing the nomogram.

Variable	Univariate logistic regression		Multivariate logistic regression	
	OR (95%CI)	P value	OR (95%CI)	P value
Gender (male vs female)	0.859 (0.419–1.762)	0.679	NA	NA
Age (year) (**<60 vs >60**)	0.714 (0.352–1.447)	0.350	NA	NA
Primary tumor site (Low-rectum VS Mid-rectum)	1.359 (0.657–2.812)	0.409	NA	NA
MRI T-stage (T1-2 vs T3-4)	0.239 (0.114–0.501)	<0.0001	0.211 (0.083–0.541)	0.001
MRI LN-status (N0 vs N1-2)	0.589 (0.272–1.273)	0.178	NA	NA
The Radiomics Score (per 0.1 increase)	2.445 (1.8–3.319)	<0.0001	2.45 (1.781–3.371)	<0.0001

Note: NA, not available. These variables were eliminated in the multivariate logistic regression model, so the OR and P values were not available.

## Discussions

This study quantified the risk probability of SLM in patients with rectal cancer by using a nomogram, and this radiomics model showed good discriminative ability to identify high risk patients with SLM. Conventional pathological predictors of SLM of rectal cancer include the primary tumor pathological grade, histological type, lymph node metastasis, invasion of the intestinal wall, and tumor markers. However, these paremeters are only available after invasive procedures, such as endoscopic biopsy or surgery. A noninvasive biomarker that can be obtained preoperatively to detect rectal cancer with SLM will be of value in clinical practice^18^. In addition, small biopsy samples may not be able to accurately reflect the global complex physiological condition of tumor^19^.

A non-invasive MRI and CT can detect SLM, and many studies had shown that MRI had a higher accuracy compared to CT in diagnosing SLM of rectal cancer patients^7,20–22^, and recent consensus guidelines were from the radiologic community recommend MRI for the preoperative evaluation of SLM^23,24^. The other studies had shown that some adverse features found on rectal MRI identified patients with rectal cancer at higher risk of distant metastasis^25–27^. Tumor stage was one of adverse features, which was closely linked to distant metastasis in patients with rectal cancer on MRI^28^. It was widely accepted that the T_2_WI sequence was the most important for preoperatively assessing Tumor stage of rectal cancer, therefore we developed a Radiomics nomogram based on T_2_WI.

We found that rad-score and mT-stage were independent predictors of SLM by univariate logistic regression. However, only a clinical factor of mT-stage in the construction of radiomics nomogram was involved by the multivariate logistic regression model, which indicated that mT-stage was closely related to SLM status. In addition, combined with mT-stage, nomograms also showed a good calibration and differentiation capabilities in training and validation sets. Of course, the importance of radiomics signature in the construction of nomograms should not be ignored. With the development of high-throughput technology and analytical approaches, multi-marker analysis had been increasingly applied. This approach combined individual markers to generate marker panels for better detection or diagnostic performance and had become an interest method in recent years^29^. For example, a prognostic classifier comprising three types of genomic and epigenomic data had improved existing protein-coding gene in lung cancer patients^30^. Similarly, the radiomics approach could incorporate individual imaging features into a radiomics signature. Meghan G *et al*. performed radiomics signatures based on T1-weighted MRI for response prediction to induct chemotherapy in patients with nasopharyngeal carcinoma^31^. Tanadini *et al*. used radiomics signature in exploratory perfusion of prostate cancer^32^, and other studies showed texture analysis of tumor response were applied in neoadjuvant chemoradiotherapy of rectal cancer^33^. These studies represented the feasibility of radiomics-based to cancer research. radiomics signatures were composed of multiple features, which was important to select optimal feature set by data dimension reduction. To our knowledge, some radiomics studies only used single method for data dimension reduction without the optimal method^14,32,34^. In this study, we investigated texture features were selected by two methods (LASSO, PCA), and we used the DCA curve to judge the optimal method for feature selection. Our results indicated that the net benefit of the LASSO method was higher than PCA. Thus, we select the method of lasso to data dimension reduction in study, which increases the stability of nomogram and hence the overall analysis.

To the best of our knowledge, this is the first study to evaluate and internally validate a radiomics nomogram composed of mT-stage and radiomics signatures to detect SLM. In a meta-analysis, gadoxetic acid-enhanced MRI (Gd-EOB-MRI) showed an excellent sensitivity of 91.2% for detecting SLM^35^. In our study, Radiomics nomogram showed a sensitivity of 73.11%. In addition, a study made by Anthony Chan *et al*.^36^ revealed the sensitivity and specificity of diffusion weighted imaging (DWI) MRI in detecting either a metastatic or indeterminate liver lesion was 87.1% and 94.5%, respectively. Although Gd-EOB-MRI and DWI MRI were more sensitive and yielded a better detection rates for SLM, we should note that the results of those study were based on the MRI examination of the upper abdomen. the cost-benefit of such an expensive and resource-consuming distinguish program was debatable. While detect SLM was based on primary rectal cancer in our study. The nomogram we constructed was easier to use for clinicians, because it could get the information from the T2WI, which could be performed quickly and contained clinical risk factors. The formal MRI with intravenous contrast was neither obligatory nor cost-effective in all patients on a rectal cancer surveillance program. The radiomics nomogram was an adjunct tool and could be used to identify and follow upon those patients with rectal cancer, which would benefit from formal MRI liver scanning. Moreover, when categorizing into low-risk and high-risk groups by the nomogram, the high-risk group had a greater probability of SLM. Therefore, in a sense, radiomics nomogram may serve as an accurate and reliable detection tool for SLM in patients with rectal cancer. It is quick and easy to perform and helpful to identify which patients will benefit from further liver imaging.

In this study, we chose the displacement vector 1,4 and 7 to calculate the GLRLM features. But when we performed feature selections, we found that the different displacement was selected out due to the high correlation (>0.9). No same GLRLM texture features with different displacements were chose in the final selected 7 features. Meanwhile, we also consult some radiomics articles and found that GLRLM features were used at the distance of 1^14,37,38^. So the use of multiple distances in GLRLM is unusual and in this study appears to be redundant.

There are several limitations in our study. First, this nomogram is based on a single institutional retrospective analysis and may not be generalized to other populations of patients with rectal cancer. Nevertheless, the analysis of this cohort enables establishment of a preliminary radiomics nomogram and facilitate future refinement of nomogram in a larger and more diverse prospective study. Second, this study lacks a external validation of the model. Multicenter validation with a larger sample size is mandatory. Thirdly, texture analysis is only performed on a single slice in our study, not covering the whole tumor volume. However, NG *et al*.^39^ showed texture analysis of largest cross-sectional area could be used as an alternative to whole tumor texture analysis. So, our research results select a single slice, which covers the largest cross-sectional area of tumor for analysis. At last, we didn’t do the gray level discretization which will influence the texture matrix calculation, we will process the gray level discretization in the following study. Despite these limitations, we hope that our experience will be contributing to accurately detect SLM. Future work will focus on validating this model.

In this study, a radiomics nomogram has been constructed by combining Tumor stage factors with radiomics signature, which facilitates the accurate detection of the probability of a patient of rectal cancer with SLM and may be helpful to physician in clinical decision.

## Supplementary information

Titile paper

## Data Availability

Data reported in this study was not available due to the limitations set by Ethical committee.
