# Supplementary material for: MRI-based Radiomics nomogram to detect primary rectal cancer with synchronous liver metastases
Source: Sci Rep. 2019 Mar 4;9:3374. doi: 10.1038/s41598-019-39651-y (PMC6399278; doi:10.1038/s41598-019-39651-y)
Supplement: Supplementary file 1 — Titile paper [file 41598_2019_39651_MOESM1_ESM.doc]

**MRI-based Radiomics nomogram to detect primary rectal cancer with synchronous liver metastases**

Zhenyu Shu^1^, Songhua Fang^1^, Zhongxiang Ding^1^, Dewang Mao^1^, Rui Cai^2^, Yuanjun Chen^3^, Peipei Pang^3^, Xiangyang Gong^1*^

*^1^Department of Radiology, Zhejiang Province People's Hospital, People's Hospital of Hangzhou Medical College, Hangzhou, China*

*^2^Department of Anorectal, Zhejiang Province People's Hospital, People's Hospital of Hangzhou Medical College, Hangzhou, China*

*^3^GE Healthcare China, Shanghai, China*

***Correspondence to:** Xiangyang Gong,

**Supplementary data**

**I. Supplementary Methods**

**1. The radiomics procedure**

**1.1 Region-of-interest segmentation procedure**

T2-weighted images (T_2_WI) were retrieved and imported into the ITK-SNAP software(www.itksnap.org) for region of interest (ROI) segmentation. A ROI was drawn around the entire tumor outline on the largest cross-sectional area of the primary tumor from the T_2_WI by a radiologist who was blinded to the clinical outcome. After that, the segmented tumor ROI files were imported into AK software (Artificial Intelligence Kit V3.0.0.R, GE Healthcare) for texture analysis.

**1.2 Data dimension reduction methodology**

Firstly, extracted texture features were standardized, which could remove the unit limits of the data of each feature so that the indexes of different units or orders could be compared and weighted. Then the feature dimensionality reduction was performed as follows. The analysis of variance (ANOVA) and Mann-Whitney U test (MW) dimensionality reduction were performed, and then the correlation test was calculated to reduce data redundancy. The software calculated the paired correlation between each two of the features. If the Spearman correlation coefficient was greater than 0.9, which showed that the two features were highly correlated, one of them was removed. All of the above steps were carried out by AK software. Figure S1 showed the flowchart of radiomics features dimension reduction. Figure S2 showed correlation analysis for each of radiomics features.

1. **Detailed descriptions of the statistical methodology and feature**
   1. **Image preprocessing**

we used z-score normalization to make the image intensities have the properties of a standard normal distribution with $\mu\text{=}1$ and $\sigma\text{=}0$, where $\mu$ was the mean value of the images, and $\sigma$was the standard deviation. The normalized values (also called z scores) of the image intensities (*x*) were calculated as follows:

$$z=\frac{x-\mu}{\sigma}$$

**2.2 Information of feature**

After z-score normalization of image pixel intensities, the number of radiomics features arrived at 328 by AK software of GE company. Radiomics features including Histogram, Formfactor, Gray-Level Co-occurrence Matrix (GLCM), Run length matrix (RLM). The details were described in the table below.

**
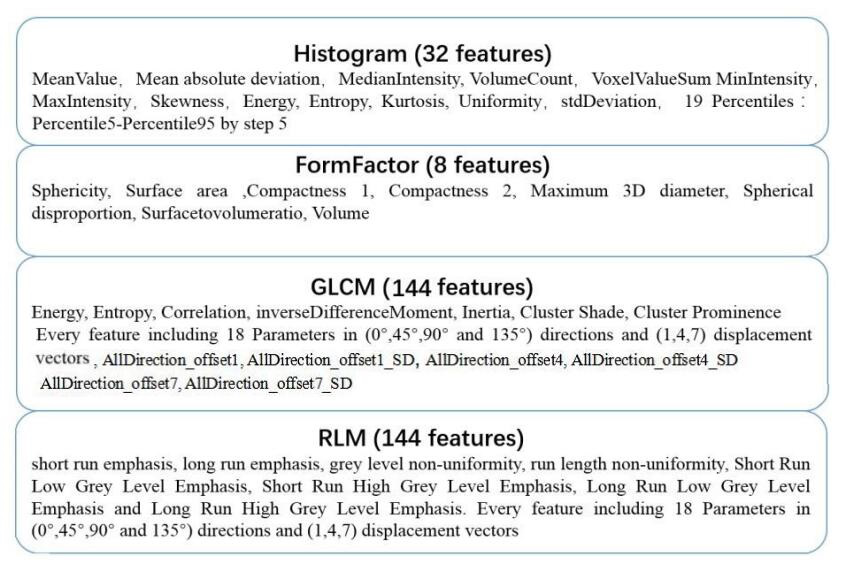
**

**2.3 The least absolute shrinkage and selection operator (LASSO) algorithm**

LASSO is a powerful algorithm for regression analysis with high dimensional predictors. In our study, the LASSO algorithm was combined with the logistic regression model for model development. We used the LASSO logistic regression model to select the most important predictive features and construct a radiomics signature in the training set. This algorithm minimizes a log partial likelihood subject to the sum of the absolute values of the parameters bounded by a constant:


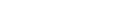
, subject to
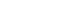


where
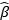
 is the obtained parameters,
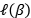
 is the log partial likelihood of the logistic regression model, and
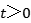
 is a constant.

The LASSO algorithm shrinks some coefficients and reduces others to exactly 0 via the absolute constraint. Thus, LASSO is an outstanding method for feature selection by retaining the good features of both subset selection and ridge regression. In this study, LASSO selected 7 nonzero coefficients
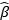
, and a formula was generated using a linear combination of selected features that were weighted by their respective LASSO coefficients. The “glmnet” package in R statistical software version 3.3.1 was used for LASSO logistic regression model analysis.

**Radiomics score calculation formula**

Rad-score = 4.19+0.009.37×GLCMEntropy_ALLDirection_offset1_SD

+0.0203×ShortRunEmphasis_ALLDirection_offset7_SD

-0.226×RunLengthNonuniformity_ALLDirection_offset4_SD

-0.0881×ShortRunEmphasis_angle35_offset7

-0.172×GreyLeveLNonuniformity_ALLDirection_offset7_SD

+0.273×GLCMEntropy_ALLDirection_offset1

-0.273×LongRunEmphasis_angle45_offset7.

Note: “SD” indicate the value reflects the standard deviation among the different directions.

**2.4 PCA**

Principal component analysis (PCA) is a statistical procedure that uses an orthogonal transformation to convert a set of observations of possibly correlated variables into a set of values of linearly uncorrelated variables called principal components. If there are *n* observations with *p* variables, then the number of distinct principal components is *min(n-1,p)*. This transformation is defined in such a way that the first principal component has the largest possible variance (that is, accounts for as much of the variability in the data as possible), and each succeeding component in turn has the highest variance possible under the constraint that it is orthogonal to the preceding components. The resulting vectors are an uncorrelated orthogonal basis set.

The steps of transformation:

1. the first step is to calculate the covariance matrix S of the matrix X

(2) the second step is to calculate the covariance matrix S eigenvector E1, E2,... EN and eigenvalues, t = 1,2,... N;

(3) in the third step, projection data is in the space formed by the eigenvector. Using the formula
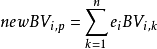
, the BV value is the value of the corresponding dimension in the original sample.

In this study, 7 features of LASSO dimensionality were analyzed by PCA, and 6 features (GLCMEntropy_ALLDirection_offset1_SD, ShortRunEmphasis_ALLDirection_offset7_SD, RunLengthNonuniformity_ALLDirection_offset4_SD, ShortRunEmphasis_angle35_offset7, GreyLeveLNonuniformity_ALLDirection_offset7_SD,GLCMEntropy_ALLDirection_offset1) contributions were greater than 85%. Therefore a regression model was performed for these 6 features to obtain the radiomics signature.

**2.5 Decision curve analysis (DCA)**

In our study, the DCA method was used to evaluate the methods of the data dimension reduction. The DCA algorithm assessed methods of the data dimension reduction by calculating the range of threshold probabilities in which a prediction or prognostic model was clinically useful.

DCA is a compositive method for evaluating and comparing different methods of the data dimension reduction. The theory of DCA can be illustrated by the equation below:

*
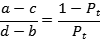
*

where *d* – *b* represents the influence of unnecessary dimension reduction. If dimension reduction is directed by a prediction model, *d* – *b* is the harm related to a false-positive result compared with a true-negative result. Inversely, *a* – *c* represents the consequence of rejecting beneficial dimension reduction, in other words, the harm from a false-negative result compared with a true-positive result. *P_t_* represents where the expected benefit of dimension reduction is equal to the expected benefit of refraining from treatment.

**2.6 Details of the GLCM and RLM**

**Co-occurrence matrices**

The Grey level co-occurrence matrix (GLCM)
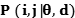
 represents the joint probability of certain sets of pixels having certain grey-level values. It calculates how many times a pixel with grey-level **i** occurs jointly with another pixel having a grey value **j**. By varying the displacement vector **d** between each pair of pixels.

The advantage of the co-occurrence matrix calculations is that the co-occurring pairs of pixels can be spatially related in various orientations with reference to distance and angular spatial relationships, as on considering the relationship between two pixels at a time. As a result, the combination of grey levels and their positions are exhibited apparently. Therefore, it is defined as “A two-dimensional histogram of gray levels for pair of pixels, which are separated by a fixed spatial relationship”. However, the matrix is sensitive to rotation. With the change of different offsets define pixel relationships by varying directions.

The rotation angle of an offset:
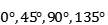
and displacement vectors (distance to the neighbor pixel: 1, 2, 3 ...), different co-occurrence distributions from the same image of reference. GLCM of an image is computed using displacement vector d defined by its radius, (distance or count to the next adjacent neighbor preferably is equal to one) and rotational angles.


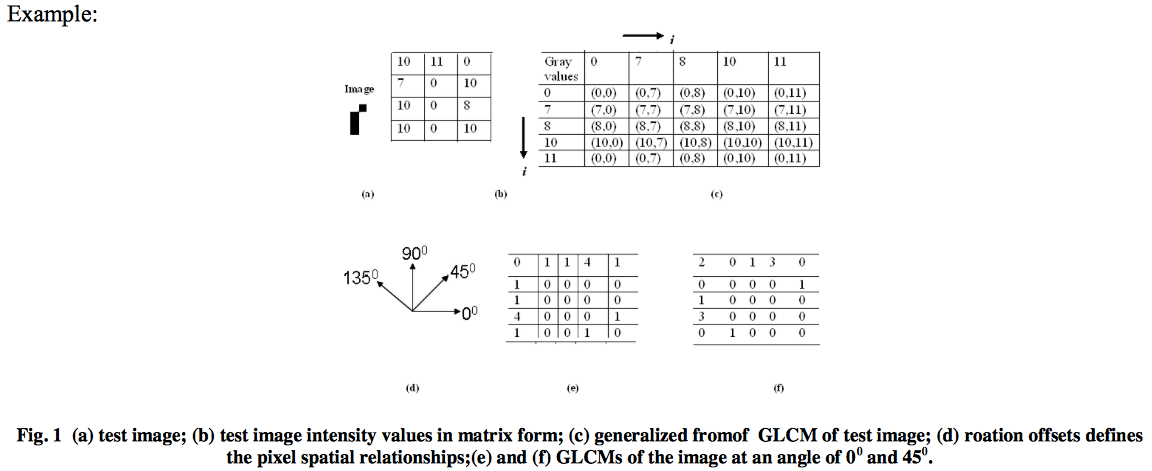


**Run-length matrices**

### The grey level run-length matrix (RLM)
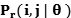
 is defined as the numbers of runs with pixels of gray level *i* and run length *j* for a given direction θ. RLMs is generated for each sample image segment having directions (0°,45°,90° &135°), then the following ten statistical features were derived: short run emphasis, long run emphasis, grey level non-uniformity, run length non-uniformity, Low Grey Level Run Emphasis, High Grey Level Run Emphasis, Short Run Low Grey Level Emphasis, Short Run High Grey Level Emphasis, Long Run Low Grey Level Emphasis and Long Run High Grey Level Emphasis.

**II. Supplementary Tables and Figure**

**Table S1. The results of data dimension reduction with LASSO and PCA.**

| Method |  | Training | validation |
| --- | --- | --- | --- |
| PCA | AUC | 0.893 | 0.735 |
|  | Specificity | 0.788 | 0.783 |
|  | Sensitivity | 0.952 | 0.778 |
| LASSO | AUC | 0.857 | 0.834 |
|  | Specificity | 0.808 | 0.783 |
|  | Sensitivity | 0.855 | 0.917 |

**Note:** AUC, area under curve; LASSO, least absolute shrinkage and selection operator; PCA, principal component analysis;

**Table S2. The classification and calculation formula of seven texture features.**

| **Category** | **Feature** | **Formula** |
| --- | --- | --- |
| GLCM | GLCMEntropy_ALLDirection_offset1_SD | 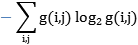  *g is a GLCM  Where i, j are the spatial coordinates of g (i,j). |
|  | GLCMEntropy_ALLDirection_offset1 |  |
| RLM | ShortRunEmphasis_ALLDirection_offset7_SD | $SRE(\theta)=\frac{1}{n_{r}}\sum_{i=1}^{M} \sum_{j=1}^{N} \frac{p(i,j,\theta)}{j^{2}}$ |
|  | ShortRunEmphasis_angle35_offset7 |  |
|  | LongRunEmphasis_angle45_offset7 | $LRE(\theta)=\frac{1}{n_{r}}\sum_{i=1}^{M} \sum_{j=1}^{N} p(i,j,\theta)j^{2}$ |
|  | GreyLeveLNonuniformity_ALLDirection_offset7_SD | $GLN(\theta)=\frac{1}{n_{r}}\sum_{i=1}^{M} (\sum_{j=1}^{N} p(i,j,\theta))^{2}$ |
|  | RunLengthNonuniformity_ALLDirection_offset4_SD | $RLN(\theta)=\frac{1}{n_{r}}\sum_{j=i}^{N} (\sum_{i=1}^{M} p(i,j,\theta))^{2}$ |

Note: GLCM, gray-level co-occurrence matrix; RLM, run length matrix

**Figure S1. The flowchart of radiomics feature dimension reduction**

**
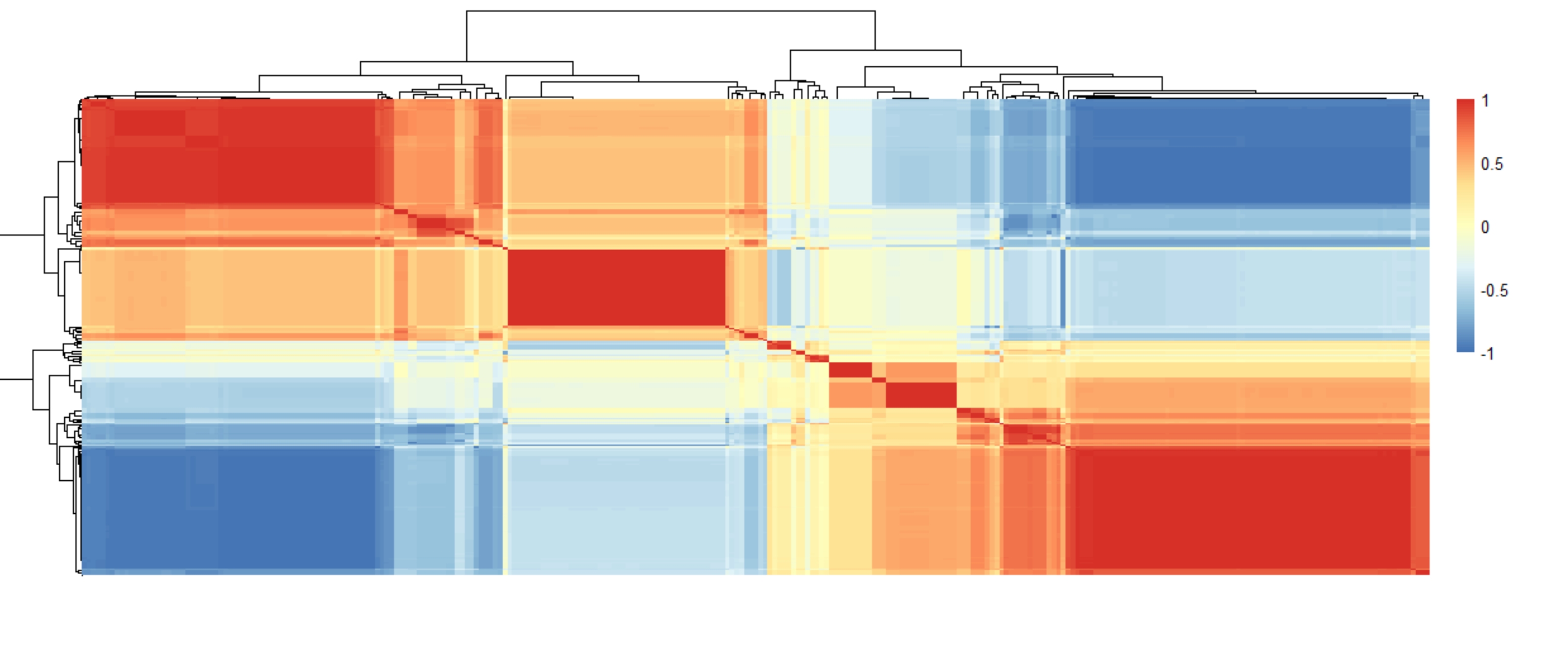
**

**Figure S2. Color mapping for the correlation analysis for each of radiomics features.** The y-axis and the x-axis presents 245 radiomics features, respectively, the y-axis on the right represents the correlation coefficient, deep Blue and deep red represents the feature showed significant association.
